# Trends and disparities in acute coronary syndrome-related mortality in the United States: Implications for healthcare

**DOI:** 10.1097/MD.0000000000044237

**Published:** 2025-09-19

**Authors:** Muhammad Khuzzaim Khan, Ibrahim Ahmed Khan, Abdul Moiz Javed, Arslan Wazir, Hira Saleem, Muhammad Zohaib, Faisal Rasheed, Priyanka Mohan Lal, Syeda Laiba Sherazi, Amiaz Karim, Nikhil Duseja, Hussam Al Hennawi

**Affiliations:** a Department of Internal Medicine, Dow University of Health Sciences, Karachi, Pakistan; b Department of Internal Medicine, Saba University School of Medicine, The Bottom, Caribbean Netherlands; c Department of Internal Medicine, Allama Iqbal Medical College, Lahore, Pakistan; d Department of Internal Medicine, University of Louisville, Louisville, KY; e Department of Internal Medicine, Ziauddin Medical University, Karachi, Pakistan; f Department of Internal Medicine, I.K. Akhunbaev Kyrgyz State Medical Academy, Bishkek, Kyrgyzstan; g Department of Internal Medicine, Al-Baath University, Homs, Syria; h Department of Internal Medicine, Karachi Medical and Dental College, Karachi, Pakistan; i Department of Internal Medicine, Jefferson Abington Hospital, Philadelphia, PA.

**Keywords:** acute coronary syndrome, age-adjusted mortality rates, geographic disparities, mortality trends

## Abstract

Acute coronary syndrome (ACS) is a major cause of morbidity and mortality in the United States (US). However, the trends and disparities in ACS mortality are not well understood. This study aimed to analyze the ACS mortality in the US from 1999 to 2020 using the Centers for Disease Control and Prevention Wide-Ranging Online Data for Epidemiologic Research database. The age-adjusted mortality rates (AAMR) due to ACS were calculated for each year from 1999 to 2020, stratified by sex, race, urbanization, and state. The trends and disparities in AAMR were examined using descriptive statistics and graphical methods. The AAMR due to ACS decreased from 1999 to 2010 and for some variables till 2018, but then increased from 2018 to 2020. The AAMR due to ACS was higher in men than in women, non-Hispanic black adults than in non-Hispanic white adults and rural than in urban populations. The AAMR due to ACS varied widely among different states, with New York having the highest AAMR and Minnesota having the lowest AAMR. This study revealed the trends and disparities in ACS mortality in the US from 1999 to 2020. The study showed an increase in AAMR due to ACS in the recent years. The study also found significant disparities in AAMR due to ACS by sex, race, urbanization, and state. Further research is needed to explore the factors that contribute to the variation and inequality in ACS mortality.

## 1. Introduction

Acute coronary syndrome (ACS), encompassing unstable angina, non-ST elevated myocardial infarction, and ST elevated myocardial infarction, poses a considerable burden of morbidity and mortality. A timely diagnosis hinges on factors such as the patient’s medical history, presenting signs, electrocardiogram findings, and troponin levels.^[1]^ The prevalence of ACS in the United States (US) is striking, affecting over 600,000 Americans annually with a daunting 40% mortality rate within 5 years for those afflicted.^[2]^

A demographic subset that confronts a substantial impact of ACS is the elderly, constituting 30% to 40% of those experiencing attacks. This group not only faces a higher mortality rate but is also susceptible to coexisting conditions such as atherosclerotic plaques, calcifications, multivessel disease, and geriatric decline, contributing to the complexity of their cases.^[3]^ Remarkably, an 8-year mortality rate of 45% in the 65 to 74 years age group and 77% in those aged 75 and above underscores the gravity of ACS in the elderly.^[4]^

Gender-related differences in ACS presentations have been noted, with variations in older age, frailty, and the impact of diabetes mellitus and hypertension. However, the statistical significance of these differences in mortality rates and prognosis remains a subject of debate. Some studies suggest no substantial difference, while others report a higher mortality rate in women, particularly in cases of acute myocardial infarction (MI) and spontaneous coronary artery dissection.^[5–8]^

Despite trials showing no disparity in mortality rates between black and white individuals with ACS, a comprehensive analysis of data involving over 49,000 patients with coronary artery disease, cerebrovascular disease, and peripheral arterial disease reveals a higher cardiovascular death rate among black individuals.^[9–11]^

Notably, a critical gap in understanding ACS mortality rates persists from 2009 to 2020, particularly concerning age, gender, ethnicity, and geographical location in the US. This study assumes paramount importance as it not only identifies disparities in the mortality rates of a treatable condition but also provides a quantitative foundation for its exploration. By doing so, it contributes to the enhancement of the healthcare system, facilitating improved patient care and treatment outcomes.

## 2. Methods

### 2.1. Study setting and population

This study delves into ACS related deaths, utilizing data spanning from 1999 to 2020. The information was sourced from the Centers for Disease Control and Prevention Wide-Ranging Online Data for Epidemiologic Research database. Analysis focused on gender, ethnicity, and geographical location within the US, utilizing the International Statistical Classification of Diseases and Related Health Problems-10th Revision codes.

The dataset comprises cause-of-death data extracted from death certificates encompassing the District of Columbia and all 50 states. This dataset, previously employed in cardiovascular disease (CVD) mortality studies, provided a robust foundation for our investigation. Our specific focus was on ACS-related deaths among individuals aged 55 years and above. For the sensitivity analysis, we broadened our scope to include ACS as a contributory factor in deaths where CVD (I00-I99) was the primary cause. All individuals aged 55 or above were considered in this analysis.

It is important to note that this study, involving secondary data analysis, falls under the exempt category and thus does not require institutional board review. All data utilized in this study were obtained from openly available resources provided by government organizations, ensuring compliance with ethical guidelines.

### 2.2. Data extraction

Our investigation involved the retrieval of data on ACS related mortality rates. Key demographic factors examined included gender, state of residence within the US, and race/ethnicity. The classification of race/ethnicity encompassed Black or African American, American Indian or Alaska Native, Whites, Hispanics or Latino, and Asian or Pacific Islander. This comprehensive approach allowed for a nuanced exploration of ACS-related mortality trends across diverse demographic groups.

### 2.3. Statistical analysis

To unveil national trends in ACS related mortality rates, we conducted a meticulous statistical examination. Crude and age-adjusted mortality rates (AAMRs) per 10,000 population were calculated for the years 1999 to 2020, stratified by year, sex, race/ethnicity, and geographical location. The calculation included 95% confidence intervals (CIs).

The AAMRs were derived by comparing ACS-related fatalities to the U.S. population in 2000, providing a robust measure adjusted for age variations. The Joinpoint Regression Program (Joinpoint V 4.9.0.0; National Cancer Institute, Bethesda, Rockville) was employed to identify the annual percent change (APC) in AAMR, offering insights into the trajectory of mortality rates over time. The statistical significance of these trends was assessed using a threshold of *P* < .05. This rigorous analysis allowed us to discern nuanced patterns and fluctuations in ACS-related mortality, providing a comprehensive understanding of its evolution across various demographic and geographic dimensions.

## 3. Results

Between 1999 and 2020, ACS emerged as a contributing factor in a substantial 8,155,995 fatalities among individuals aged 55 years and older. This toll was distributed across genders, with 4,289,212 males and 3,855,784 females affected.

Examining the spatial context of these fatalities, data on the location of death was available for 7,813,535 cases. Notably, 29.42% of these occurrences transpired in the familiar setting of home, while 23.48% unfolded within nursing homes or other long-term care facilities. A smaller proportion, 1.96%, took place in hospices, while the majority, accounting for 45.12%, occurred within the confines of medical institutions.

### 3.1. Annual trends for ACS-related AAMR

The average AAMR associated with ACS during the extensive period of 1999 to 2020 was 511.0 (95% CI: 510.7–511.4). Notably, the overall ACS-related AAMR exhibited a consistent and noteworthy decline, starting at 823.2 in 1999 and reaching a considerably lower value of 377.1 by the conclusion of 2020.

Over the years, the APC in ACS-related AAMR depicted distinctive phases. Between 1999 and 2002, there was a pronounced negative trend with an APC of −3.92 (95% CI, −5.04 to −2.16). Subsequently, from 2002 to 2010, this decline intensified, showcasing an APC of −5.56 (95% CI, −6.80 to −5.15). However, from 2010 to 2018, a shift occurred, and the APC slightly increased to −2.81 (95% CI, −4.06 to −2.34). Notably, in the most recent years, from 2018 to 2020, the APC experienced a modest ascent to 0.87 (95% CI, −1.70 to 2.3).

### 3.2. ACS-related AAMR stratified by sex

**Figure 1.**
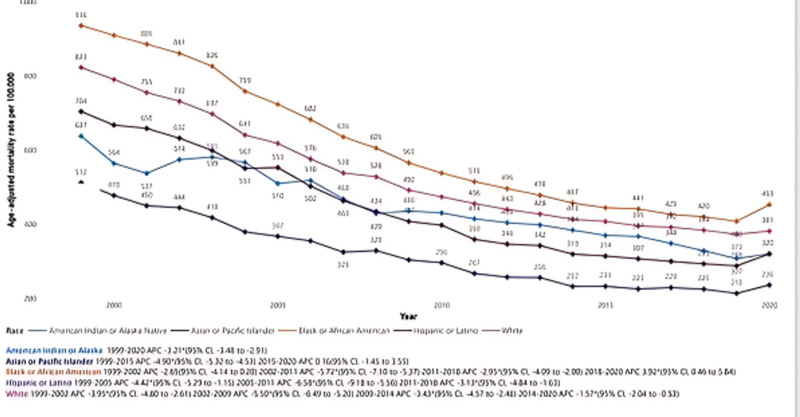
Trends in acute coronary syndrome-related age-adjusted mortality rates (AAMR) from 1999 to 2020, stratified by gender.

Between 1999 and 2020, a discernible pattern emerges when stratifying AAMR by gender. Males consistently exhibited higher AAMR compared to females, a trend vividly illustrated in Fig. 1, which also reflects a gradual decrease in AAMR for both genders during this period.

For males, the AAMR underwent a substantial decline from a formidable 1046.6 (95% CI: 1042.3–1051) in 1999 to 513.7 (95% CI: 511.5–516) in 2020. Conversely, females experienced a decrease from 666 (95% CI: 663.3–668.6) in 1999 to 270.2 (95% CI: 268.8–271.6) in 2020. On average, the AAMR for males was 661.0 (95% CI: 660.3–661.6) and for females, it was 399.1 (95% CI: 398.7–399.5).

Exploring the APC in AAMR for males between 1999 to 2002 revealed a negative trend of −3.66 (95% CI: −4.92 to −1.68), deepening from 2002 to 2007 to −5.76 (95% CI: −7.10 to −4.22). Subsequent years saw a rise in APC from 2007 to 2014, reaching −3.62 (95% CI: −4.33 to −2.20). Between 2014 to 2020, the APC for males experienced a further ascent to −1.11 (95% CI: −1.76 to 0.22).

For females, the APC from 1999 to 2002 was −3.93 (95% CI: −5.06 to −1.91), intensifying from 2002 to 2011 to −5.96 (95% CI: −7.38 to −5.69). The subsequent years displayed a gain in APC from 2011 to 2018, reaching −3.34 (95% CI: −5.50 to −2.71). The period between 2018 to 2020 witnessed a climb in APC to 0.03 (95% CI: −2.81 to 1.76).

### 3.3. ACS-related AAMR stratified by race

**Figure 2.**
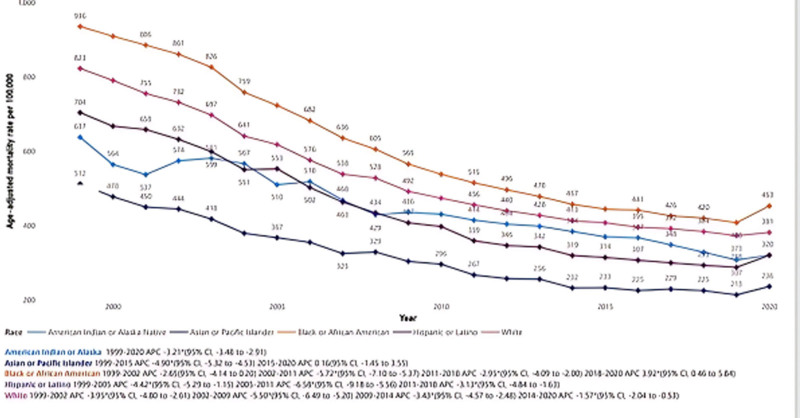
Acute coronary syndrome-related age-adjusted mortality rates (AAMR) stratified by race/ethnicity (1999–2020).

In Fig. 2, the stratification of AAMR by race/ethnicity reveals distinct patterns in ACS-related mortality trends across diverse racial groups. The average AAMR from 1999 to 2020 was highest among Black or African American individuals (581.8; 95% CI: 580.5–583.1), followed by White individuals (519.8; 95% CI: 519.4–520.2), American Indian or Alaska Native individuals (422.5; 95% CI: 417.7–427.2), Hispanics or Latinos (398.3; 95% CI: 397.1–399.5), and Asian or Pacific Islander individuals (285.7; 95% CI: 284.3–287.2). Black or African American AAMR declined from 935.6 (95% CI: 926.7–944.4) in 1999 to 452.6 (95% CI: 448–457.1) in 2020. White individuals saw a reduction from 822.6 (95% CI: 820–825.1) to 381.4 (95% CI: 380–382.9). American Indian or Alaska Native individuals experienced a decline from 637.4 (95% CI: 602.1–672.7) to 320.2 (95% CI: 305.1–335.3). Hispanics or Latinos showed a decrease from 703.8 (95% CI: 693.1–714.5) to 319.9 (95% CI: 315.9–323.9), and Asian or Pacific Islander individuals decreased from 511.8 (95% CI: 498.3–525.3) to 236.0 (95% CI: 231.4–240.5). APC trends further delineate these differences: Black or African American individuals experienced APCs ranging from −2.65 (95% CI: −4.14 to 0.20) in 1999 to 2002 to a notable increase of 3.92 (95% CI: 0.46 to 5.84) in 2018 to 2020. For White individuals, the APC shifted from −3.95 (95% CI: −4.80 to −2.61) in 1999 to 2002 to −1.57 (95% CI: −2.04 to −0.53) in 2014 to 2020. American Indian or Alaska Native individuals maintained a relatively steady downward trend with an overall APC of −3.21 (95% CI: −3.48 to −2.91) from 1999 to 2020. The Hispanic or Latino population experienced a steeper decline in certain periods, with the most marked drop from 2005 to 2011 at −6.58 (95% CI: −9.18 to −5.56). Asian or Pacific Islander individuals exhibited a consistent decline from 1999 to 2015 with an APC of −4.90 (95% CI: −5.32 to −4.53), followed by a plateau between 2015 and 2020, with an APC of 0.16 (95% CI: −1.45 to 3.55).

### 3.4. ACS-related AAMR stratified by age group

Age-stratified AAMR analysis was conducted for individuals aged 55 years and older. The results demonstrated that individuals aged 85 and older had the highest average AAMR, at 2879.6 (95% CI: 2878.0–2881.3), followed by those aged 75 to 84 with an AAMR of 1211.4 (95% CI: 1210.2–1212.5), those aged 65 to 74 with an AAMR of 582.7 (95% CI: 581.9–583.4), and those aged 55 to 64 with an AAMR of 272.5 (95% CI: 271.8–273.2). Across all age groups, AAMRs showed a declining trend from 1999 until approximately 2018, followed by a plateau or slight increase through 2020. The steepest relative declines were observed in the 65 to 74 and 75 to 84 age groups, although the 85+ population consistently carried the highest mortality burden throughout the study period. These findings reinforce the disproportionate impact of ACS on older adults and emphasize the need for targeted age-specific preventive strategies.

### 3.5. ACS-related AAMR stratified by geographical region

The geographical variation in AAMR for ACS unfolds a diverse landscape, highlighting substantial differences from state to state. New York emerges with the highest AAMR at 699.4, while Minnesota records the lowest at 309.6. Notably, 8 out of 51 states exhibit AAMR above 600, encompassing New York and Oklahoma. Another 15 states fall within the range of 500 to 600, including California, Florida, and Pennsylvania. The remaining states report AAMR below 400.

Breaking down the regional averages, Northeast America registers an average AAMR of 567.6 (95% CI: 566.8–568.4), the Midwest reports 516.3 (95% CI: 515.5–517), the South notes 502.6 (95% CI: 502–503.2), and the West reports 462.9 (95% CI: 452.1–463.6).

A noteworthy trend surfaces when comparing metropolitan and nonmetropolitan areas. Metropolitan areas exhibit a slightly lower AAMR, with a decrease from 808.85 in 1999 to 367.925 in 2020. In nonmetropolitan areas, AAMR declines from 822.5 in 1999 to 429.6 in 2020. The APC in metropolitan areas declines from −3.79 (95% CI: −4.76 to −2.08) in 1999 to 2002 to −5.77 (95% CI: −6.85 to −5.43) in 2002 to 2009. Between 2009 to 2016, the APC increases to −3.40 (95% CI: −4.20 to −2.78), further rising to −0.78 (95% CI: −1.76 to 1.20) from 2016 to 2020. Conversely, in nonmetropolitan areas, the APC is −4.45 (95% CI: −4.76 to −4.19) between 1999 to 2011, which decreases to −1.53 (95% CI: −1.97 to −0.96) from 2011 to 2020.

**Figure 3.**
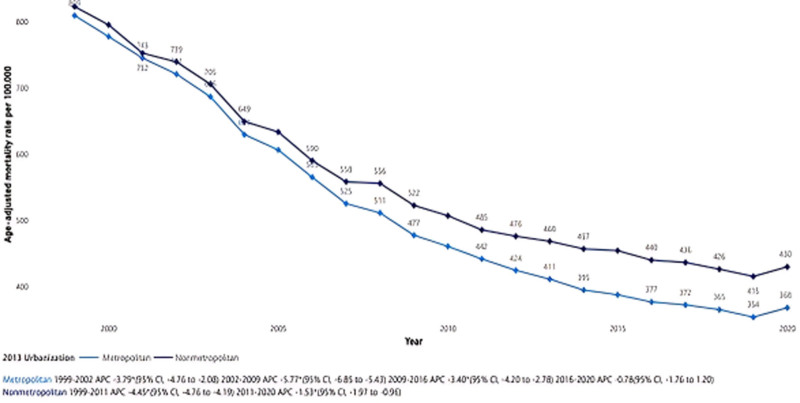
Geographical trends in acute coronary syndrome-related age-adjusted mortality rates (AAMR) from 1999 to 2020.

Figure 3 visually captures these regional and metropolitan/nonmetropolitan trends, providing a comprehensive overview of the intricate geographical dynamics influencing ACS-related AAMR.

## 4. Discussion

This study analyzed ACS mortality trends in the US from 1999 to 2020 using the Centers for Disease Control and Prevention Wide-Ranging Online Data for Epidemiologic Research database. The findings revealed 2 distinct temporal patterns: a steady decline in AAMRs from 1999 to 2010 (and up to 2018 for certain subgroups), followed by an upward trend from 2018 to 2020. These findings are consistent with prior research on acute MI mortality, which also reported a recent increase using data up to 2019.^[12]^ Our inclusion of 2020 data extends these observations and emphasizes the resurgence of ACS-related mortality in recent years.^[13,14]^

In terms of sex-based disparities, our study found that AAMRs due to ACS were consistently higher in men than in women. This contrasts with several earlier studies that reported higher mortality in women following acute MI.^[15,16]^ The discrepancy may be partially explained by differing pathophysiological mechanisms, with women often experiencing atypical or nonobstructive coronary artery disease, which may not be classified under ACS in administrative databases. Furthermore, women may present later to care, receive less aggressive treatment, or be underdiagnosed. However, once care is accessed, men may experience more severe ACS phenotypes with higher short-term fatality, potentially explaining the higher overall AAMRs in men observed here.

Regarding age-related trends, our study focused on adults aged 55 years and older, revealing that this group carries a significant burden of ACS mortality. Previous studies have shown relatively stable CVD mortality rates among younger adults aged 25 to 54, but increasing rates among those aged 55 to 64 years from 2011 to 2017.^[13]^ Similarly, Dani et al reported higher CVD mortality in middle-aged adults (55–64) compared to younger groups, although their analysis only included data through 2019 and excluded individuals over 65 years.^[13]^ Our study not only incorporates data for 2020 but also includes individuals beyond 65 years, revealing that ACS mortality is substantially higher in the elderly, with prior studies reporting 8-year post-MI mortality rates of 47% in patients aged 65 to 74 and 77% in those 75 and older.^[14]^ This underscores the need for intensified preventive and post-discharge care in older populations.

In terms of racial disparities, non-Hispanic (NH) Black adults exhibited the highest AAMRs, followed by NH White individuals, with notable fluctuations observed among American Indian or Alaska Native populations between 1999 and 2005. These findings are consistent with prior evidence that NH Black Americans have higher prevalence of cardiovascular risk factors such as hypertension, diabetes, obesity, and albuminuria.^[17–19]^ Additionally, structural inequities in healthcare access, socioeconomic barriers, and differences in health-seeking behaviors may exacerbate these disparities. Blackstone et al attributed poorer outcomes in NH Black adults to worse pre-MI health status and unfavorable MI characteristics, including smaller infarcts, higher likelihood of heart failure during hospitalization, nonspecific electrocardiogram findings, and reduced rates of coronary revascularization.^[20,21]^ These mechanisms may underlie the persistently elevated mortality observed in our study.

Our analysis also showed urban–rural disparities, with rural populations exhibiting higher AAMRs than urban ones. This pattern aligns with previous studies^[22,23]^ and may reflect multiple systemic challenges: limited access to cardiologists, differences in emergency medical services capabilities (often staffed by volunteers in rural settings), disparities in treatment approaches between generalists and specialists, and inadequate prehospital care. Moreover, although rural residents may attend cardiac rehabilitation sessions at slightly higher rates than their urban counterparts,^[24]^ barriers such as travel distance, fewer health facilities, and socioeconomic hardship may diminish the overall benefit. For example, a study from Nebraska demonstrated that patients who attended post-MI cardiac rehabilitation had significantly lower mortality than those who did not.^[25]^

Finally, state-level disparities were prominent, with New York reporting the highest AAMR (699.4) and Minnesota the lowest (168.9). These geographic differences may be driven by variations in healthcare infrastructure, quality of care, public health policies, socioeconomic status, and lifestyle behaviors such as smoking, diet, and physical activity. Further research is warranted to explore the root causes of such variability and to design region-specific interventions.

## 5. Conclusion

This study unveils distinct shifts in ACS mortality trends, emphasizing a concerning rise post-2019. Gender and racial disparities challenge established norms, demanding further exploration. Geographic variations underscore the need for targeted interventions. The findings prompt ongoing vigilance and adaptive healthcare strategies to address the evolving landscape of ACS mortality.

## Author contributions

**Conceptualization:** Arslan Wazir, Hussam Al Hennawi.

**Data curation:** Ibrahim Ahmed Khan, Abdul Moiz Javed, Faisal Rasheed, Syeda Laiba Sherazi.

**Formal analysis:** Ibrahim Ahmed Khan, Abdul Moiz Javed, Hira Saleem, Muhammad Zohaib, Faisal Rasheed, Priyanka Mohan Lal, Syeda Laiba Sherazi, Nikhil Duseja.

**Investigation:** Muhammad Khuzzaim Khan, Hira Saleem, Muhammad Zohaib, Priyanka Mohan Lal, Nikhil Duseja, Hussam Al Hennawi.

**Methodology:** Muhammad Khuzzaim Khan, Hira Saleem, Muhammad Zohaib, Priyanka Mohan Lal, Nikhil Duseja, Hussam Al Hennawi.

**Project administration:** Muhammad Khuzzaim Khan, Arslan Wazir, Amiaz Karim, Hussam Al Hennawi.

**Resources:** Arslan Wazir, Amiaz Karim.

**Software:** Ibrahim Ahmed Khan, Abdul Moiz Javed, Faisal Rasheed, Syeda Laiba Sherazi.

**Validation:** Hira Saleem, Muhammad Zohaib, Priyanka Mohan Lal, Nikhil Duseja.

**Visualization:** Ibrahim Ahmed Khan, Abdul Moiz Javed, Faisal Rasheed, Syeda Laiba Sherazi.
